# Seeing the unseen: a theory of sexual misconduct perception in the surgical workplace

**DOI:** 10.1093/bjs/znag077

**Published:** 2026-06-16

**Authors:** Rebecca A Fisher, Philippa C Jackson, Greta McLachlan, Carrie Newlands, Christopher T Begeny, Rosalind Searle

**Affiliations:** Behavioural Science for International Health Workforce Research Group, University of Manchester, Manchester, UK; Department of Surgery, North Bristol NHS Trust, Bristol, UK; Department of Surgery, Frimley Health Foundation Trust, Frimley, UK; School of Biosciences and Medicine, University of Surrey, Guildford, UK; Faculty of Health and Life Sciences, Department of Psychology, University of Exeter, Exeter, UK; Adam Smith Business School, University of Glasgow, Glasgow, UK

## Abstract

**Introduction:**

Sexual misconduct in surgery is recognized as widespread, yet individuals within the same departments report markedly different perceptions of prevalence. Quantitative data show gender differences in experiences, but reasons for this remain unclear. This study explored perceptions of sexual misconduct in the UK surgical workplace and developed a heuristic framework.

**Methods:**

Qualitative analysis was undertaken of free-text responses from 742 participants in a 2022 nationwide UK survey. Respondents included students, trainees and consultants. Inductive, reflexive thematic analysis within a constructionist paradigm was conducted, with iterative coding by two researchers and team discussions to refine themes and develop an explanatory framework.

**Results:**

Accounts diverged. Some participants described respectful workplaces with little misconduct, whereas others reported pervasive harassment, assault and inaction. Using an X-ray metaphor, the ‘Lateral View X-ray’ theory was developed to explain this. The ‘AP View’ reflects unawareness or minimization, sustained by limited exposure, normalization as banter, defensiveness, and seniority-related distancing. The ‘Lateral View’ reflects heightened awareness from personal or witnessed experiences, hypervigilance, frustration at inaction, and informal warning networks. Male victims described additional barriers to recognition and disclosure. Drawing on standpoint and epistemic injustice theory, this work demonstrates how social position, power, and language shape whether misconduct is recognized or obscured.

**Conclusion:**

The Lateral View X-ray theory explains how sexual misconduct can be simultaneously pervasive and invisible within surgery. Awareness depends on vantage point, where those least exposed perceive little problem, whereas those affected perceive structural harm. Embedding this framework in surgical education may widen perspective, improve responses and support cultural change.

## Teaser

Ever seen the lateral view X-ray and seen something you couldn't see before? We conceptualize a new understanding of sexual misconduct in the surgical workplace to aid future training.

## Introduction

Sexual misconduct is increasingly recognized as a serious problem in medicine, particularly in the surgical workplace. In 2022, this team conducted the largest UK study to date, revealing that 63.3% of women surgeons reported being the target of sexual harassment, and 29.9% reported sexual assault in their career^1^. Among men, prevalence was markedly lower (23.7% and 6.9%, respectively). Fewer men reported that they had witnessed sexual harassment (81% *versus* 89.5%, *P* < 0.001) or sexual assault (17.1% *versus* 35.9%, *P* < 0.001) than women colleagues in the same workplace.

These findings, consistent with other international studies, showed differences in perception of workplace culture. Although quantitative findings established the scale of the problem, they did not examine divergence in perception between groups. This study used the large qualitative data set to explore these findings, with the aim of exploring perceptions of sexual misconduct in the surgical workplace. Furthermore, to identify factors contributing to awareness or unawareness and to develop a conceptual framework to explain these differences and inform education and cultural change.

## Methods

### Study design and paradigm

A qualitative analysis of free-text survey responses was conducted within a subjectivist epistemology, acknowledging that participants interpret, rather than objectively observe, their world. A constructionist paradigm underpinned the study, with experiences and meanings understood as socially produced through context and cultural norms. Analysis was inductive, generating an explanatory framework from participants’ accounts rather than testing predefined hypotheses.

### Ethical considerations

This study received HRA/HCRW approval (Health Research Authority/Health and Care Research Wales; reference 22/HRA/3738) and ethical clearance from the University of Exeter’s Faculty of Health and Life Sciences Psychology Research Ethics Committee (Reference 511842). Data were anonymized prior to analysis by a non-medical team member (C.B.) followed by a second round by a surgeon (C.N.).

### Context and participants

Data were drawn from a nationwide cross-sectional survey conducted between 17 January and 27 February 2022. The survey was distributed via the Association of Surgeons of Great Britain and Ireland, the Royal Colleges of Surgery, surgical specialty associations, and professional networks. Eligible participants were individuals working or training in UK surgical settings, from medical students to consultants. Of 1704 respondents, 742 provided free-text responses; 51.5% were women, 63.1% consultants, and 20.2% specialty trainees. Full demographics are reported in the authors’ previous study^1^.

### Data collection

The survey included items on demographics, role, and experiences of sexual misconduct, alongside optional free-text fields. Prompts invited descriptions of personal and witnessed experiences and reflections on surgical workplace culture. All questions were optional, and participants indicated consent for publication of quotes.

### Analysis

Free-text responses were imported into NVivo (v15) for analysis. All 742 participants were analysed but only the 241 who consented to publication were quoted. Sample size was determined by the prior quantitative study. The granular survey design and large sample yielded information-rich data with sufficient information power to develop new concepts^2^. Reflexive thematic analysis followed Braun and Clarke’s six phases^3^ (*Box 1*).

Box 1 A summary of reflexive thematic analysis using Braun & Clarke’s 6 phases

**Phase 1: Familiarization with data**

Transcripts were read by two authors (C.B., C.N.) during the prior quantitative arm of this study. An initial observation was made that men and women were living in ‘different worlds’.

**Phases 2–4: Generating initial codes, generating themes and reviewing themes**

Two researchers (R.F. and R.S.) independently conducted line-by-line coding on an initial subset to develop a preliminary codebook. Coding was latent and iterative, with coding discrepancies resolved through reflexive dialogue at regular team meetings. These discussions were used to refine the developing coding framework, forming broader themes that captured shared patterns across the data set. Negative case analysis was conducted by returning to the data to search for experiences that contrasted with dominant patterns. These findings were used to challenge assumptions and refine the developing theory.

**Phase 5: Defining and naming themes**

Through reflexive team discussions with all authors, these themes were distilled into a unifying metaphor—the ‘Lateral View X-ray’—that acts as a heuristic framework to explain the divergence in perceptions.

**Phase 6: Producing the report**

Preliminary results were presented at the Professional Standards Authority Research Conference 2024 and the International Conference on Residency Education 2025, where feedback was used to shape this report.

**Reflexivity**

The analysis team comprised six researchers, including four female surgeons, one male academic psychologist and one female professor in human resource management and organizational psychology. Several team members had prior research or lived experience relating to gender equity and harassment in surgery^4,5^. Reflexive discussions were held throughout the project to examine how these positions and assumptions might shape interpretation.

## Results

Overall, 1703 free text responses were made by 742 participants. All responses were analysed and quotations are from the 241 participants who consented to publication. Participants gave a mean 6.4 responses, with a median 85 words in total (i.q.r. 35–190). A minority provided long responses (maximum 2080 words total).

**Figure 1:**
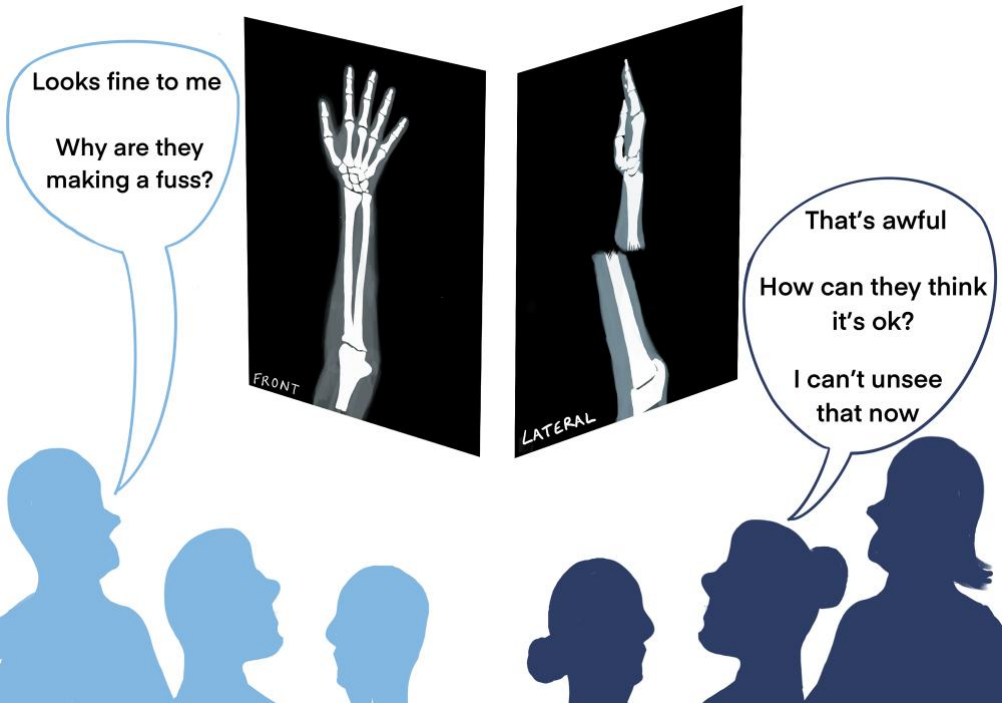
The Lateral X-Ray metaphor. The ‘AP’ view is shown on the left and the ‘lateral view’ on the right. Original image created by Rebecca A. Fisher. Alt text: Two groups of people are looking at two black and white X-rays of a forearm and hand. On the left, the arm looks normal. There is a speech bubble that says looks fine to me, why are they making a fuss? On the right, the x-ray shows an arm that is clearly broken. The people look at it and are saying that′s awful, how can they think that′s ok? The two images and groups are separated so the other group cannot see the other image.

Participants’ accounts diverged where for some, sexual misconduct seemed endemic, for others it was scarcely perceptible. Institutional variation was not supported as an explanation as both perspectives co-existed within the same settings, suggesting divergence arose from differing vantage points. Several interpretive lenses were explored, including trauma- and resilience-informed approaches, before developing a framework aligned with the data. Using an X-ray metaphor, the framework illustrates how the same workplace may appear intact from one angle yet ‘broken’ from another. Two groups were identified: (1) those unaware of misconduct (‘AP View’), whose perspective resembles an anteroposterior X-ray that can miss hidden fractures, and (2) those aware and vigilant (‘Lateral View’), recognizing cultural ‘damage’ unseen by others (*Fig. 1*).

### The AP View

Participants with the AP View described workplaces as respectful or free from misconduct. Where acknowledged, misconduct was seen as rare, isolated, or trivial. This perspective reflected limited exposure, normalization as banter, dismissive attitudes towards allegations, and a distancing through seniority, reducing personal targeting and suggesting the ‘problem’ had diminished over time.

#### Lack of exposure

Some respondents attributed their AP View to not being present when misconduct occurred, describing long careers without witnessing serious incidents.

[I have spent] forty years as an NHS consultant … I do not recall any incident which I would believe would today be categorized as sexual assault or sexually motivated in a pathological sense.

I did not see any evidence of sexual harassment, assault or rape. Although there was some evidence of misogynistic behaviour towards women […] where I worked as a consultant, which could be classed as a form of sexism this was not sexually motivated.

#### Normalization

Others acknowledged witnessing inappropriate behaviour, but framed it as ‘harmless banter’, particularly in theatre teams where sexualized jokes were common and accepted.

I regard [sexual jokes/banter] as normal human behaviour.

… there still is normal interaction that can involve jokes with a sexual element that does not offend people who know each other and work together.

Sexual jokes and comments are honestly so common in my workplace. All of the instances I have witnessed or been the victim of have had straight male perpetrators. They seem to have no idea that it is unacceptable.

A male colleague was giving a presentation and was heckled by another male colleague asking him to ‘undo another button’ [then] asked whether this should be a button on his shirt or his trousers. […] It was seen as a joke between friends, but sets a poor example of tolerance for disrespect for those who are vulnerable to sexual perpetrators …

A consultant used to rub past my breast in theatre when reaching for particular things and would announce out loud ‘mind your boobs’. There was enough room there was no need to do that. Everyone in the room could see what he was doing and often the staff on the floor would laugh.

… we were talking about plans for the evening. I said I still felt quite hyped up and probably needed to go for a run or lift some heavy weights; the consultant then responded with ‘it sounds like you need to get railed through a headboard’. This was whilst still operating.

#### Denial or defensiveness

Some participants dismissed misconduct allegations as being overblown, framing them as ‘over-policing’ of normal workplace interactions.

Again the perpetrator self-excused their behaviour saying, ‘I’m just a very tactile person’ and ‘I guess I’m not supposed to do this anymore’.

#### Seniority

Respondents noted that career stage shaped experiences. Senior consultants, particularly women, perceived less misconduct directed towards them, potentially due to reduced targeting.

It is usually senior male consultants who have always behaved this way, and they usually have power over their own and other specialties.

If I [woman consultant] said anything other than laughing it off and, now that I am more senior/respected, saying something like ‘come on now …’, I suspect I’d be slowly freezed out of research and training.

A senior anaesthetist: … although I like to think the culture is changing, I do worry that it is just becoming less overt. [This is good but] it is harder to be an ally if you don’t actually see it happen. I also suspect that as I am now senior and possibly more immune from anyone else being able to influence my career.

A small number rejected that misconduct occurred in their workplace, while supporting taking it seriously if it did.

Some discussion, talk and ‘jokes’ are within the realm of orthodox human discussion. To believe otherwise is rather daft. It is when someone is directly denigrated or threatened by the talk and actions that it becomes a serious matter worth reporting.

The NHS is becoming an increasingly unpleasant place to work with PC gone mad. The heavy-handed policing of individuals for telling ‘off’ jokes will result in an increasingly Stalinist workplace. Where there is serious sexual misconduct, it should be dealt with by the police.

### The Lateral View

In contrast, those with the Lateral View described acute awareness of misconduct, often grounded in personal experience or repeated witnessing. Accounts reflected both exposure to harassment or assault and heightened vigilance for cues. They were frustrated at colleagues who remained unaware or failed to act. Informal ‘whisper networks’ warned others about perpetrators. Some male participants described harassment by women colleagues, highlighting additional gendered complexities.

#### Personal exposure

For many, their awareness came from direct harassment or assault.

There are jokes of a sexual content most days at work. Yesterday, [a male peer] made a comment about the size of his own erection. […] I’ve become used to this sort of ‘banter’ and it’s rarely directed at me which makes it easier to tolerate but it is unfortunately part of my day-to-day job.

I was afraid to go in the lift due to constant groping from multiple members of the team. […] On one occasion I sat between two male colleagues at audit meeting who each put a hand up my dress at the same time. I was horrified but their hands met & they both started shouting at each other […] I was later reprimanded by team lead for upsetting them!

Wanted sexual favours in return for signing a form/signing me off. [They are] still working with vulnerable people.

#### Hyperawareness

Some respondents described their constant vigilance to misconduct cues, shaped by personal trauma or repeated exposure.

My daughter was raped (not in healthcare) […] She has complex PTSD from this. […] It means I am more easily upset by what may be seen by others as light-hearted innuendo etc.

I am particularly concerned about high-risk settings e.g. conferences with overnight stays, works parties, off site social events where alcohol and different grades of staff mix.

One of my colleagues used to become inappropriate with female trainees when drunk. […] We used to distract him and move him away, […] We were always vigilant for this type of behaviour at work social events.

#### Frustration at others’ ignorance or inaction

Vigilant participants often expressed anger towards colleagues who remained in the AP View, and frustration at organizational inaction.

I was told that my complaint was not the first about [them, and] not to refer to it as sexual harassment as this could be classed a defamation of the consultant’s reputation which could be used against me legally.

We had an SHO that was sexually harassing F1s. He did it to multiple women and they were really distressed. We reported it and after an initial meeting with the perpetrator the trust only properly investigated it a year later. It was appalling.

#### Whispered warnings

In some cases, those holding the Lateral View formed informal networks designed to protect others that is pre-warning colleagues about known perpetrators and advising them how to be avoided.

Throughout my career there have been known senior doctors who have the reputation for being ‘handsy’ and female trainees warn each other about them. This behaviour seemed to be tolerated or excused by their colleagues.

I had been warned about this consultant’s behaviour by other female trainees before starting the job. [One] female trainee that complained about him was blacklisted and not trained. All behaviour warned about happened: back/neck massages, hip thrusting during laparoscopic surgery, ‘fixing’ his mask on your shoulder, ‘accidental’ boob graze.

He is known as inappropriate with female trainees. Female trainees are told they will get good operating numbers if they go in his theatre because he will train them if they can ‘cope with his behaviour’. He has never been disciplined for this.

#### The Lateral View for male victims

Although most respondents described male perpetrators, some men reporting harassment identified women colleagues, often senior theatre nurses. Normalization of sexual banter made such behaviour more accepted and less likely to be reported.

Open discussion during theatre case about my penis size and sexual habits—I was the only man in the theatre.

Good looking male registrar quite often gets unwanted sexual attention from nursing and healthcare staff with inappropriate groping on occasion.

On one occasion I spoke up in theatre and said that if men were doing this to a young woman, they would be sacked. So why did they think this was OK? […] Despite how he handled it publicly, I suspect it took its toll as he left the profession after about a year of this treatment.

Some male participants also described feeling constrained in responding to women perpetrators, believing that the #MeToo movement made counter-complaints riskier for them as a man.

She made her intentions known on more than one occasion […] She then made comment about me to a senior colleague, citing a number of occasions when I had been sexually inappropriate towards her […] I listened to their advice that as the man in the time of the ‘me too’ culture that this would be seen as ‘there is no smoke without fire’.

## Discussion

This study is likely one of the largest qualitative data sets on sexual misconduct in surgery. In-depth enquiry was limited by the non-interactive survey design. This had epistemological implications, as the authors could not reflexively adapt questions or co-produce theory. The anonymous design, however, also lent strength, capturing voices otherwise silenced due to the oppressive and complex nature of sexual misconduct. Many participants shared longitudinal accounts, with a minority providing in-depth responses. It is likely that those with strong experiences, particularly victims, were more likely to contribute and have their experiences amplified. Although this was anticipated, the authors were struck by the other common perspectives, including indifference or resistance to the suggestion of misconduct. This stark divergence in perceptions triggered theory development.

The Lateral View X-ray theory shows how awareness depends on vantage point that is those in privileged positions may have a narrow ‘AP View’ that misses the problem, while those with lived experience gain a ‘Lateral View’ that makes misconduct visible and impossible to ignore. As a clinician, one learns the habit of looking for an alternative X-ray view, and it is suggested here to apply this habit to the workplace culture. Practising this is essential for those at lower risk of witnessing misconduct, such as men and senior staff.

Although male victims are highlighted, this issue is driven by gendered power asymmetries, with leadership more often male and victims and witnesses more often female. Standpoint theory describes how those in marginalized positions, such as women and junior staff, occupy a social standpoint that provides clearer insight into structural problems than those in privileged roles^6,7^. In surgery, junior women are often more attuned to subtle misconduct and cultural hostility, whereas senior men, buffered by status, may remain unaware. This framework helpfully adopts a no-blame stance, recognizing how an individual may hold an ‘AP View’ despite good intentions. Gendered differences in workplace perception emerge early in medical school. In one study, men accounted for 72.3% of active participation in teaching in a cohort of 44.3% men. When asked, men were much less likely to be aware of this social imbalance^8^. Sometimes, people are unable to fully recognize or describe what has happened because the shared social concepts needed to interpret the experience are missing or incomplete. Philosophers call this epistemic injustice, which helps explain why misconduct remains hidden^9^. Failures to recognize or ‘hear’ reports involves ‘pluralistic ignorance’ thus ‘making it difficult to know what is in their interest to know’^10^. One form, hermeneutical injustice, arises when concepts or language to make sense of an experience are missing or underdeveloped^11^. Before the 1970s, the term ‘sexual harassment’ did not exist, making it difficult for victims to name or convey their experience. A similar blind spot, or hermeneutical gap, may persist for subtle behaviours that fall short of assault yet cause harm. Nielsen *et al*.^12^ showed participants often reserved the label ‘sexual harassment’ for overt, coercive, or physical misconduct, while dismissing subtler behaviours as merely ‘unwanted attention’. Recognizing an incident as harassment was often a social process, shaped by peer validation and cultural norms. As Searle notes, those subjected to misconduct may take time to acknowledge it, while perpetrators are often in denial^13,14^. Movements such as #MeToo have played a pivotal role, providing language and social legitimacy to reframe ambiguous experiences as harassment thus a shift from the AP View to the Lateral View. Until all healthcare workers understand the social factors enabling misconduct, many may, however, remain in the AP View—not out of denial, but because they cannot interpret events as misconduct.

The Lateral View X-ray metaphor offers a powerful educational tool based on a simple clinical concept. It simplifies a continuum of awareness into two categories, that cannot capture the full complexity of lived experiences but may prompt reflection through its oversimplicity. Trainers can use it to help employees recognize the limits of their perspective and broaden understanding in remedial and active bystander training. This may improve institutional culture and professional performance. The tool could also prompt reflective practice for progression and revalidation, and the authors hope this framework will be tested and refined in clinical settings.

The Lateral View X-ray theory offers a novel metaphor to explain how sexual misconduct can be simultaneously pervasive, and invisible in surgery. Changing perceptions requires no-blame education to improve recognition of sexual misconduct, listening to marginalized voices, and creating safe reporting channels. As in clinical practice, seeking the lateral view is essential to make the hidden visible.

## Data Availability

Quantitative data underlying the findings in this article are available at the Center for Open Science (https://osf.io/q4th5/). To preserve participant anonymity, this publicly accessible version of the data does not contain any free-text responses from participants, and some information has been aggregated or redacted to avoid potential identification of individual participants.

## Subject

Education
